# Application of Spectrofluorimetry to Evaluate Quality Changes in Stored Blue Honeysuckle Berry (*Lonicera kamtschatica*) Preserves

**DOI:** 10.3390/molecules30051012

**Published:** 2025-02-22

**Authors:** Joanna Banaś, Magdalena Michalczyk, Marian Banaś

**Affiliations:** 1 Department of Biotechnology and General Technology of Food, Faculty of Food Technology, University of Agriculture in Krakow, Balicka 122, 30-149 Krakow, Poland; magdalena.michalczyk@urk.edu.pl; 2 Department of Power Systems and Environmental Protection Facilities, Faculty of Mechanical Engineering and Robotics, AGH University of Kraków, A. Mickiewicza 30, 30-059 Krakow, Poland; mbanas@agh.edu.pl

**Keywords:** honeysuckle berry, phenolic compounds, anthocyanins, spectrofluorimetry, multidimensional analysis, PCA, LDA

## Abstract

The aim of this study was to use a rapid and non-invasive spectrofluorimetric method to evaluate the qualitative changes occurring in stored Kamchatka berry preserves. Honeysuckle berries were preserved by freezing (−24 °C) and pasteurisation with and without sugar addition. Pasteurised samples were stored at 6 ± 1 °C and 22 ± 1 °C for 9 months. During storage, spectrofluorimetric spectra in the bioactive compounds’ fluorescence range were registered. The obtained synchronous spectra were used in a statistical analysis involving principal component analysis (PCA) and linear discriminant analysis (LDA). The analysis of both types of registered spectra indicated that sugar addition could stabilise some phenolic compounds, like gallic acid, p-coumaric acid, and phloridzin. Moreover, some differences in the degradation rate of each analysed compound were observed depending on the preservation method used. Besides the phenolic compounds, other fluorescent compounds like B-vitamins and chlorophyll forms were also observed. Pasteurisation caused the distinct degradation of protochlorophyll forms, whereas practically no changes in the amounts of vitamins B_3_ and B_9_ were observed. Based on the results of statistical analyses of PCA and LDA, the effect on the products’ composition was moderate for the storage time and relatively low in the case of the storage temperature. The obtained results indicated that spectrofluorimetry would be a useful method for the detailed characterisation of fruit products.

## 1. Introduction

Blue honeysuckle berry (*Lonicera caerulea* L. var. *Kamtschatica*) is a valuable plant that is relatively easy to grow, frost resistant, has an early fruiting season and tasty fruit, and grows best at high altitudes and in colder climates [1]. Besides the name blue honeysuckle, such terms as sweet berry honeysuckle, honeyberry, haskap berry, or edible honeysuckle can be found in the literature [2].

Honeysuckle berries are a good source of biologically active compounds like anthocyanins, which can have antioxidant, antimicrobial, antidiabetic, neuroprotective, and cardiovascular effects [3]. The total anthocyanin content in fresh fruit varies from 150 mg/100 g f.w. to 655 mg/100 g f.w., depending on the variety [4]. According to Grobelna et al. [5], the content of anthocyanins ranges from 400 to 1500 mg/100 g. The main compound from this group is cyanidin-3-*O*-glucoside. Other minor anthocyanins identified in honeysuckle berries are cyanidin-3,5-diglucoside, cyanidin-3-*O*-rutinoside, peonidin-3-*O*-glucoside, and pelargonidin-3-*O*-glucoside [6]. Oszmiański et al. [7] also have reported the presence of malvidin-3-*O*-glucoside and cyanidin-3-*O*-gentobioside. According to Gorzelany et al., the content of cyanidin-3-*O*-glucoside in two varieties of honeysuckle berries is equal to 375 and 382 mg/100 g [8].

Besides anthocyanins, honeysuckle berries contain relatively high amounts of other phenolic compounds. Polak et al. [9] reported that in 100% juice of honeysuckle berries, the total polyphenol content was 308.3 mg/100 mL, whereas other authors showed values that varied from 163.0 mg/100 mL [10] to 1019.7 mg/100 mL [11]. The difference in the polyphenol content depends on the cultivar, cultivation conditions, post-harvest treatment, and technological process used. Compounds from this group identified in the examined fruits were hydroxycinnamic acids like chlorogenic acid, neochlorogenic acid, and caffeic acid derivative. At lower concentrations, there were the flavonols quercetin-3-rutinoside, quercetin-3-glucoside, quercetin-3-galactoside, and quercetin [12].

Molina et al. [13] found five organic acids (mainly citric acid 2.8 g/100 g f.w.) and at lower concentrations malic acid (0.8 g/100 g f.w.) and quinic acid (0.4 g/100 g f.w.), four free sugars (mainly fructose and glucose, total of 7.9 g/100 g f.w.), α- and γ-tocopherol (total of 0.9 mg/100 g f.w), and 20 fatty acids (mainly linoleic acid). The vitamin C content in these fruits is relatively low; values found in the literature are up to 30 mg/100 g f.w. [13,14,15,16].

In addition to polyphenols, the fruit also contains iridoids. Depending on their structure, iridoids may exhibit various biological activities, including anti-inflammatory effects and reducing the risk of metabolic diseases. However, secoiridoids have a stimulatory effect on the secretory function of the digestive glands and are known for their bitterness [7]. Iridoids are rarely found in fruit. Among others, loganic acid, 7-epi-loganic acid, 7-epi-loganic acid 7-*O*-pentoside, 8-epi-loganic acid, loganin, secologanin, secoxyloganin, taxifolin 7-*O*-dihexoside, taxifolin 7-*O*-hexoside, pentosyl sweroside, and sweroside have been found in honeysuckle berries [4,7,17]. According to Kucharska et al. [4], the total iridoid content in 30 different cultivars ranged from 120 mg/100 g f.w. to 273 mg/100 g f.w. Oszmiański and Kucharska [7] note that these properties make honeysuckle berries a good ingredient for dietary supplements and medicinal products.

Despite their attractiveness, which is connected with the contents of the compounds mentioned above, fresh honeysuckle berries are hardly commercially available. This is due, among other things, to the fact that they are harvested in a relatively short period; the fruit is delicate and has a short shelf life. Hence, preserves made from it would be much more accessible to consumers. Although there has been considerable interest in the composition and potential use of honeysuckle berries for health improvement [12,18] and food colouring [13], the effect of processing on their components has not been widely researched.

One of the barriers to be overcome when assessing the influences of processing and storage on quality changes in processed fruit products is the need for time- and cost-consuming research. It, therefore, seems useful to seek methods that will allow the effects of individual processing modifications to be analysed more simply and less expensively while at the same time reducing the negative influence on the environment. Such a method can be spectrofluorimetry, which enables the determination of different groups of compounds during one unit measurement, which is difficult using chromatographic methods. This technique demonstrated higher sensitivity than other spectroscopic tools, with a quantification limit reaching 1 ppb and being simple, reliable, and economical [19]. Moreover, it is possible to measure fluorescence from the raw material sample without pretreatment. This method was successfully used to study several food matrices, such as vegetable oils, milk and dairy products, wine, cereals, beverages, etc. [20,21,22,23,24]. To date, the spectrofluorimetric analysis has not yet been used to evaluate honeysuckle berry preparations. It is also difficult to find papers concerning the implementation of fluorescence spectroscopy to assess the influences of processing and storage on the quality of berry products. However, Sikorska et al. [23] used this technique to characterise the fluorescence of commercial beverages of chokeberry, strawberry, raspberry, and blackcurrant. These previous studies highlight the applicability of fluorescence-based methods in food quality assessment, further supporting our choice of spectrofluorimetry for evaluating quality changes in honeysuckle berry preserves.

In the present study, spectra obtained using both excitation–emission spectrofluorimetry and synchronous spectrofluorimetry techniques were analysed. The difference between the two techniques consists, among others, of using a single excitation wavelength in the former case and a selected range of excitation wavelength in the case of synchronous spectrofluorimetry [25]. Due to the high complexity of the obtained spectrofluorimetric spectra, they are usually subjected to statistical analysis using chemometric methods of pattern recognition: unsupervised methods like principal component analysis (PCA) and supervised methods such as linear discriminant analysis (LDA) [26].

The aim of the study was the implementation of the spectrofluorimetric techniques to determine the influences of preservation methods and storage conditions on bioactive substance contents in honeysuckle berry preservatives. PCA and LDA methods were used to explain changes that occurred during the preservation and storage of the analysed samples.

## 2. Results and Discussion

### 2.1. Fluorescence Spectra

As mentioned earlier, finding data related to the use of spectrofluorimetry in honeysuckle berry research in the literature is difficult. These fruits contain high amounts of polyphenols [5,9,11]. Amongst this complex group of compounds, there are a number that are fluorised. Besides polyphenols, other fluorophores, such as vitamins B_3_, B_6_, and B_9_ and protochlorophylls, chlorophylls, and their derivatives, were detected.

#### 2.1.1. Emission Spectra in the Wavelength Range of 340–550 nm

**Figure 1:**
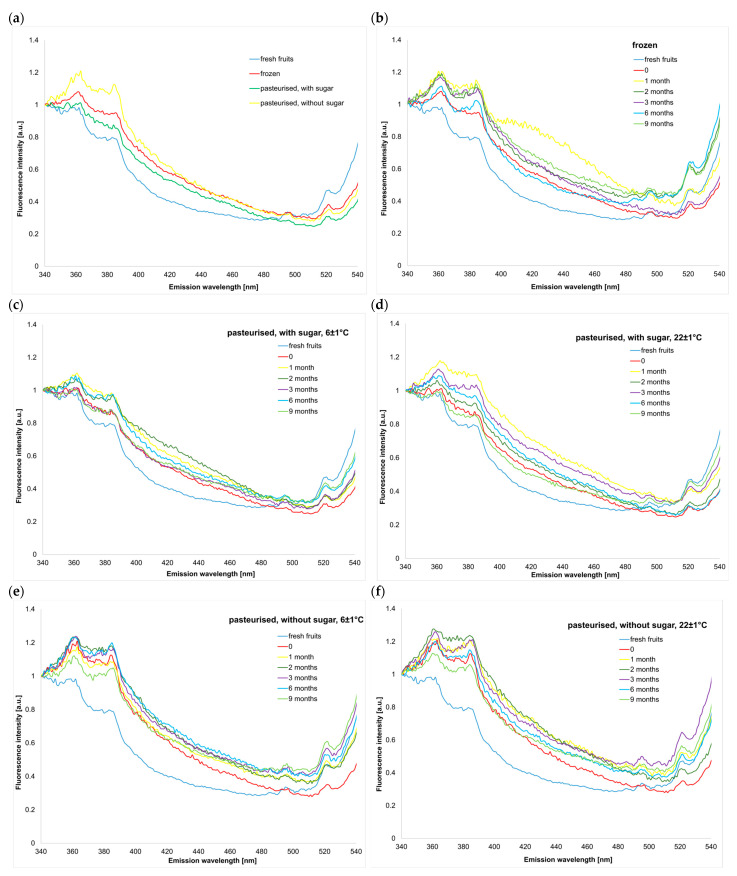
Emission spectra in the wavelength range of 340–550 nm of honeysuckle samples: after preservation (**a**); frozen during storage (**b**); pasteurised with sugar, stored at 6 ± 1 °C (**c**); pasteurised with sugar, stored at 22 ± 1 °C (**d**); pasteurised without sugar, stored at 6 ± 1 °C (**e**); and pasteurised without sugar, stored at 22 ± 1 °C (**f**).

The easiest technique to characterise samples using fluorescence spectroscopy is to record one excitation or emission spectrum. Emission spectra in the emission wavelength range of 340–550 nm (λ_ex_ 280 nm), presented in Figure 1, according to the literature data, enable the observation of phenolic compound fluorescence [27,28,29].

For systems containing several fluorophores, the signal is the result of the signals of all fluorescing substances in a given spectral range. Moreover, the signal intensity can also be affected by factors such as potential quenching and existing interactions with the environment, such as solvent or temperature effects [24,30]. These interactions can also cause shifts in the positions of the fluorescence intensity maxima of individual bands, as can be seen in the data available in the literature [22,23,28,29,30,31,32,33,34].

The maximum observed in the spectra presented in Figure 1a–f at ca. 360 nm can be ascribed to the fluorescence of substances such as gallic acid, syringic acid, and vanillic acid. The next band with a maximum of 390 nm represents the fluorescence of 3-hydroxybenzoic acid, ellagic acid, and naringenin [31]. This range is also ascribed to fluorescence of vitamin B_6_ forms [32]. The broad band with a relatively low intensity at ca. 420 nm can be attributed to the presence of 2-hydroxycinnamic acid, 4-hydroxybenzoic acid, p-coumaric acid, and ferrulic acid. This spectral range is also related to the fluorescence of niacin (vitamin B_3_) [33]. A low-intensity band with a maximum at 460 nm evidenced the presence of compounds such as chlorogenic acid, caffeic acid, rutin, and folic acid (vitamin B_9_) [31,34]. The last of the bands seen in the presented spectra with a maximum of 520 nm represents the fluorescence of quercetin and phloridzin.

The preservation method used influenced, to varying extents, the changes in the contents of the identified compounds and their mutual relations in the products analysed (Figure 1a). Generally, an increase in fluorescence intensities at 360 and 390 nm was observed, with the latter being relatively greater. Bands located at 420 and 460 nm only slightly increased in intensity, whereas, in the band at 520 nm, some decrease was observed compared to the fresh fruit spectrum. The most significant change was found in the pasteurised sample with sugar addition.

Changes caused by storage conditions in the amounts of fluorophores analysed in the present spectral range are seen in Figure 1b–f. The storage of samples pasteurised without sugar addition at 6 ± 1 °C (Figure 1c) caused relatively small changes in the fluorescence intensity of each band. Considering the individual group of compounds ascribed to them, no significant differences in their degradation dynamics were observed. Similarly, samples stored at a higher temperature (22 ± 1 °C) also did not show the aforementioned differences; only more significant changes in the intensity of the bands analysed at each storage stage were seen (Figure 1d).

Sugar addition to the honeysuckle berries before pasteurisation probably caused the stabilisation of some polyphenols present in the investigated products. The observed changes in fluorescence intensity varied to different extents for each of the analysed bands, and they were more distinct in the case of samples stored at 22 °C.

The most significant differences in the fluorescence intensity of the frozen product (Figure 1b) were observed after 1 month of storage, especially of the band at 420 nm. The following months caused only some fluctuation of the registered intensities of bands at 360, 380, 420, and 460 nm. Some differences can be observed in the case of the intensity of the band at 520 nm.

According to the literature data [35], honeysuckle berries contain hydrocinnamic acids and derivatives (30.40–156.20 mg/100 g), like p-coumaric acid, as main parts of phenolic compounds. Gallic, chlorogenic, caffeic and ferulic acid, rosmarinic and vanillic acids were also found, but their amounts were much lower. Rutin and quercetin were also present at 8.2–10.1 mg/100 g and 13–41 mg/100 g, respectively [35].

Thus, the main literature data concern the presence and contents of particular phenolic compounds in honeysuckle berries and related products, and the range of total polyphenol content changes during storage under different conditions [1,2,3,9]. However, it is relatively difficult to find research results concerning the changes in individual phenolic compound contents during storage. In the case of anthocyanins, the range of changes in the contents of the most relevant anthocyanins present in the mixed honeysuckle berry–black chokeberry juices was examined using chromatographic methods, and it was found that they degraded over practically the same range, with no evident differences in the dynamics of this process [9]. Spectrofluorimetry enables the analysis of qualitative changes occurring in the fluorescent phenolic compounds in blue honeysuckle berries. A quantitative analysis using fluorimetric methods is also possible after the extraction of the raw material and after the calibration curves have been drawn for the standards. However, the potential loss of information from the product matrix and the influence of the solvent used on the position and shape of the recorded bands must be taken into account here when performing analyses using extracts. On the other hand, determining individual fluorophores without prior extraction is connected with considering the possible quenching by other components present in the sample [24,30].

While spectrofluorimetry provides a rapid and effective tool for assessing qualitative changes in bioactive compounds, its full potential can be realised when it is combined with complementary chromatographic or mass spectrometric methods for a detailed quantitative analysis.

#### 2.1.2. Synchronous Spectra

Synchronous fluorescence spectroscopy (SFS) is based on simultaneously scanning a range of excitation and emission wavelengths with a constant difference, Δλ = λ_em_ − λ_ex_, between them. The Δλ values are chosen to obtain a favourable narrowing and separation of the bands and to minimise scattered radiation. It is very useful for investigating complex mixtures of fluorophores because both emission and excitation data are included in a single spectrum. Its advantage over the total luminescence technique is the simplification of the spectra and the rate at which they are obtained [36]. The simplification of the spectra results from preferential enhancement of the strong bands [21]. The resulting spectra provide detailed information on the presence and composition of fluorescent compounds, such as polyphenols, flavonoids, and other secondary metabolites. SFS is particularly useful for analysing food products, as it can reveal changes caused by processing methods (e.g., freezing and pasteurisation) and storage conditions.

**Figure 2:**
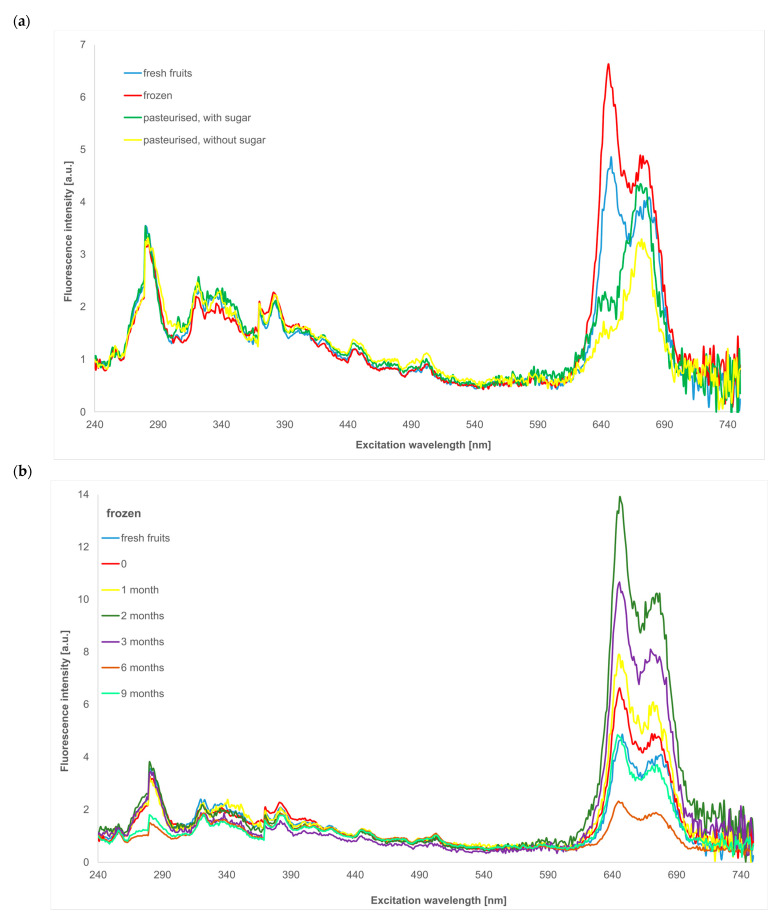
Synchronous spectra in the excitation wavelength of 240–750 nm for Δλ = 40 nm of honeysuckle samples: after preservation (**a**) and frozen during storage (**b**).

The Δλ = 10–60 nm range with a step of 10 nm was used in the present studies. Synchronous spectra recorded for Δλ = 10 nm were characterised by numerous narrow bands, almost non-overlapping, with a relatively high intensity, and rather difficult to interpret. Along with the increase in the assumed wavelength difference, the broadening of the bands and decreases in their intensities and numbers were observed. Ali et al. reported that Δλ = 60 nm is optimal to obtain the appropriate intensity of individual fluorescence bands and make the interpretation of the spectra easier [29]. Because of the easier analysis of the observed bands, spectra obtained for Δλ = 40 nm were chosen, and they are presented in Figure 2.

Figure 2a shows synchronous spectra registered for fresh fruits and samples just after preservation methods were used, such as pasteurisation with and without sugar addition and freezing.

The low-intensity band at 260 nm can be ascribed to the fluorescence of phloridzin, and the amount of this compound practically does not change during preservation processes. The next band with a maximum at 282 nm and shoulder at shorter wavelengths is related to the fluorescence of caffeic acid (λ_exc_ 270 nm), vanillic acid (λ_exc_ 278 nm), catechin and epicatechin (λ_exc_ 278 nm), and probably rutin, for which the maximum of excitation is placed at around 300 nm [31,32,37]. The addition of sugar stabilised caffeic acid; its fluorescence intensity did not change after pasteurisation, but in the case of samples pasteurised without sugar and frozen, the amount of this compound decreased (at the same range). A similar pattern was observed for the rest of the compounds fluorised in this wavelength range.

The next analysed band even contains three peaks in the excitation wavelength range of 315–360 nm. This range is ascribed to the fluorescence of such compounds as gallic acid (λ_exc_ 320 nm), p-coumaric acid (λ_exc_ 350 nm), the second band of phloridzin (λ_exc_ 330 nm), and forms of vitamin B_6_ (λ_exc_ 315–340 nm) [31,32]. The most significant changes in this range were observed after freezing. Sugar addition stabilised all fluorophores in this spectral range, whereas pasteurisation without sugar caused a slight degradation of p-coumaric acid.

The fluorescence band with a maximum at ca. 390 nm, as seen in the excitation wavelengths range of 360–440 nm, is related to the presence of ferulic acid (λ_exc_ 370 nm), quercetin, and its derivatives (λ_exc_ 410, 420 nm) [31]. Pasteurisation with and without sugar addition did not cause distinct changes in the contents of the compounds mentioned above. Some not-so-important changes were seen after the freezing of honeysuckle berries.

Two low-intensity bands located in the ranges of 440–470 nm and 490–530 nm indicated the presence of vitamin B_9_ and B_3_, respectively [33]. Some degradation of these substances was observed after freezing, whereas after both types of pasteurisation, their amounts were almost the same.

The last analysed fluorescence band in the presented spectra has a higher intensity and is related to chlorophylls and their derivatives (maximum at ca. 670 nm) [20]. It is also seen in the band at a shorter excitation wavelength (at ca. 650 nm), which, according to the literature data, can be ascribed to the fluorescence of protochlorophylls and their derivatives [38,39]. Freezing of honeysuckle berries does not change chlorophyll forms in the plant material; the observed differences in their amount are probably the result of the cellular structure transformation caused by low temperature. Homogenisation and heating used to prepare the pasteurised samples caused the substantial degradation of protochlorophyll forms, which was more distinct for samples with sugar. The amount of chlorophyll forms was almost the same in the sample pasteurised with sugar compared to fresh fruits, whereas some decrease could be observed for the sample without sugar.

Because of the complexity of the obtained spectra and the dominant role of the fluorescence intensity of chlorophylls form bands, storage-induced changes in the fluorophores mentioned in the earlier part are shown in the example of a stored frozen product (Figure 2b).

The distinct fluorescence intensity changes can be seen in the range of caffeic acid, vanillic acid, catechin, epicatechin, and rutin fluorescence. Three months of storage practically did not change the amounts of these compounds, whereas the rest of the analysed period was connected to a substantial decrease in band intensity. A gradual decrease in fluorescence intensity was also observed in the wavelength range related to the presence of ferulic acid and quercetin and its derivatives.

During the whole storage time, both protochlorophyll and chlorophyll forms were present. Their amounts changed distinctly, mainly because of the mentioned earlier transformations of the cellular structure of products.

An analysis of synchronous spectra for each type of preservation and during storage can provide valuable information on product quality or enable the early detection of the degradation of bioactive components in the product. A similar usage of these spectra to evaluate storage and heat treatment-induced changes in oils can be found in the literature [20], where chemometric methods, similar to those used in the current study, have been used to more accurately analyse subtle changes in the registered spectra.

#### 2.1.3. PCA and LDA

Three SFS spectra were collected for each sample, resulting in more than 500 measurement points (from 240 nm to 750 nm in 1 nm steps) for six different Δλ values. Registration of the synchronous spectra for each type of sample (fresh, frozen, and pasteurised) for the following storage steps under different conditions and six various storage times (from 0 to 9 months) generated a large dataset that required advanced analytical techniques for proper interpretation. For this purpose, principal component analysis (PCA) and linear discriminant analysis (LDA) were applied.

**Figure 3:**
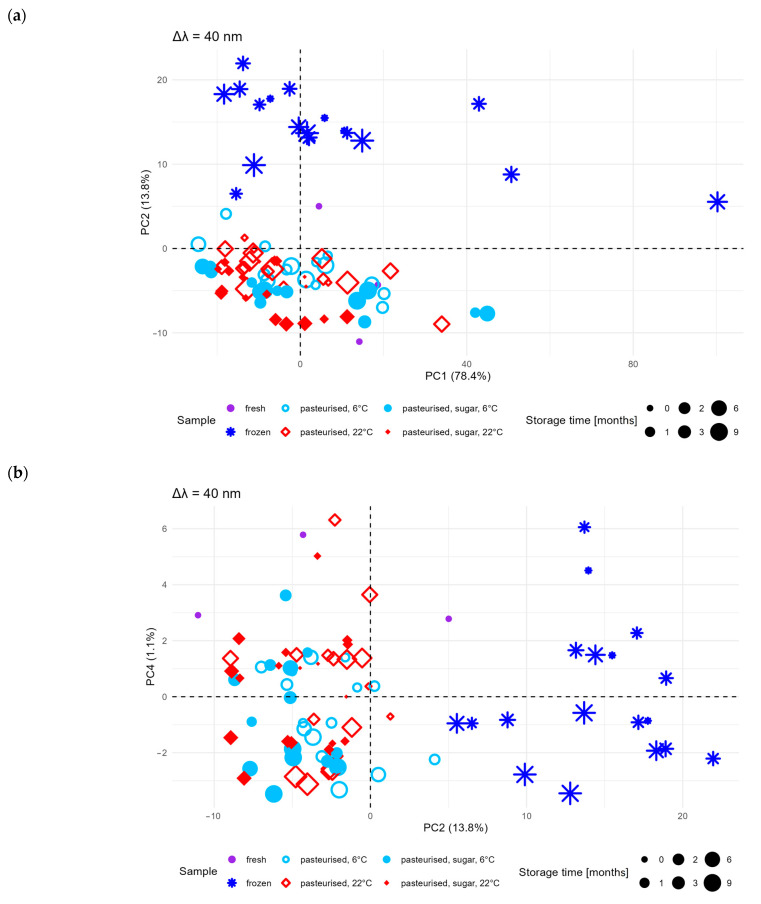
Scatter plot of the first two principal components (PC1 and PC2) (**a**); scatter plot of the second and fourth principal components (PC2 and PC4) (**b**).

The main purpose of this interpretation was an exploratory analysis of the results to indicate the existence of the influence of selected parameters (type of preservation, sugar addition, storage conditions, and Δλ value) and to classify the individual spectra using the factors indicated as discriminating criteria for these samples. The PCA (principal component analysis) algorithm, in addition to its main application of dimensionality reduction (so that instead of several hundred features, only a few new features, called principal components, can be used), also allows the classification of individual measurements by clustering them on principal component planes (PCA1:PCA2, etc.) [26]. It transforms the original variables into a new principal component set that maximises the explained variance. The results of the analysis are presented in Figure 3. As can be seen, the PC1 and PC2 components explained 78.4% and 13.8% of the total variance, respectively.

On the PC1:PC2 plane (Figure 3a), the frozen berry samples were clearly separated from the others, indicating significant differences in their fluorescence characteristics. Little separation of samples pasteurised with sugar from those without sugar addition was also found. There was no tendency to discriminate between samples stored at 6 ± 1 °C and at 22 ± 1 °C. Samples stored at different times (in the range of 0 to 9 months) did not create separate groups.

The obtained results showed that time of storage had no significant effect on the changes observed in the spectra, whereas the addition of sugar during pasteurisation had little effect. On the PCA2:PCA4 plane (Figure 3b), the pasteurised samples with and without sugar were more distinguishable; however, these components (PC2 and PC4) explained only a small portion of the total variance (13.8% and 1.1%, respectively). This suggests that some class-discriminating information is captured in less dominant components.

However, while PCA effectively reduces the dimensionality of the data, in some cases, it does not provide complete class separation, as was the case for samples stored at different temperatures (6 °C and 22 °C). In this case, although the method identified important differences (e.g., between frozen and fresh samples), it did not guarantee the complete separation of samples based on all parameters, such as the storage time or temperature. Some differences may have been missed because PCA does not always detect subtle changes in less dominant components. In addition to the fact that this technique allowed a significant proportion of the variability in the data to be captured, as PCA1 and PCA2 explained 78.4 % and 13.8% of the total variance, respectively, the components with smaller contributions to the variance (PCA3 and PCA4) may contain important but more difficult to interpret information. When analysing pasteurised samples with and without sugar, PCA showed some differences, but these were less pronounced, suggesting that the results obtained may not be entirely conclusive in such cases.

Using PCA enabled a preliminary exploration of the spectral data structure, identifying significant differences, particularly for frozen and pasteurised samples. Although it did not fully separate all classes, it proved valuable for dimensionality reduction and detecting overall trends.

Because the principal component biplots did not separate effectively into clusters showing the influence of selected parameters (storage conditions and type of preservation), a supervised classification technique, i.e., with the assumption of partitioning into selected classes, was used to discriminate the samples with an algorithm that was much more effective at classification compared to PCA, namely, linear discriminant analysis. The input dataset for LDA was the same as for PCA.

**Figure 4:**
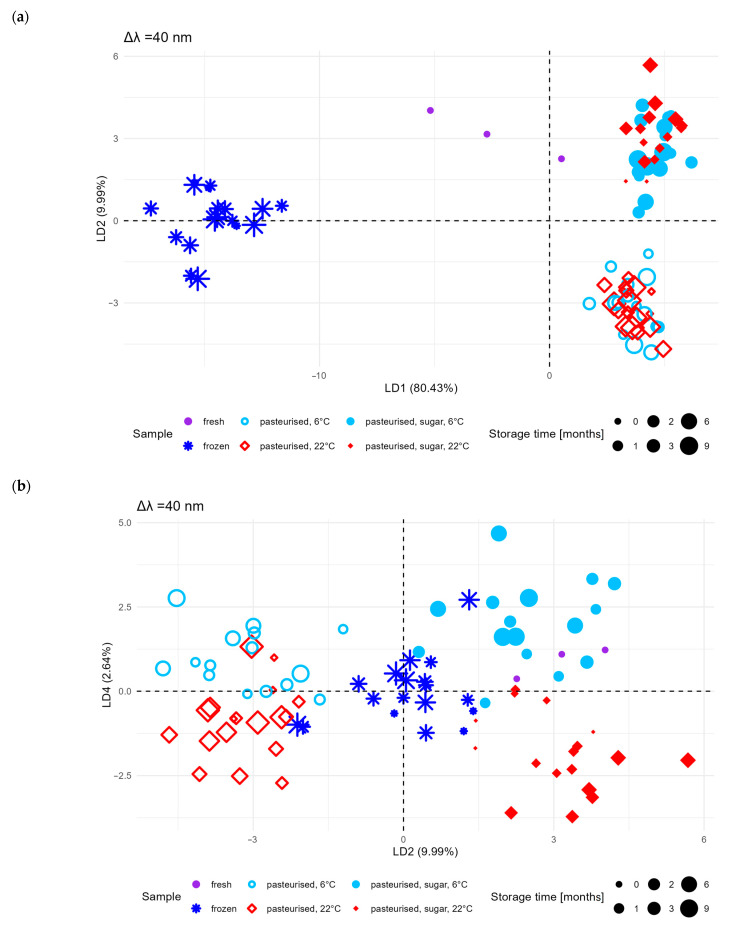
Scatter plot of the first two linear discriminants (LD1 and LD2) (**a**); scatter plot of the second and fourth linear discriminants (LD2 and LD4) (**b**).

This technique maximises the separation between groups by finding linear combinations of variables that best separate the classes. The application of LDA allowed a better separation of sample groups (Figure 4). The contributions of the subsequent discriminant functions to the explained variance were LDA1 80.43%, LDA2 9.99%, and LDA4 2.64%, respectively. A clear separation between fresh and frozen fruit samples was observed on the LDA1:LDA2 plane (Figure 4a). There was also a clear separation between pasteurised samples without sugar and pasteurised samples with sugar. However, there was still no clear distinction between samples stored at different temperatures (6 and 22 °C). On the other hand, by analysing the LDA2:LDA4 plane (Figure 4b), a clear separation between the points from samples stored at the two temperatures mentioned could be observed.

Despite the better separation of the groups using LDA, a clear differentiation between samples stored under refrigeration (6 ± 1 °C) and at room temperature (22 ± 1 °C) on the LDA1:LDA2 plane was still not obtained. This suggests that the effect of storage conditions is less significant than that of pasteurisation and sugar addition.

LDA assumes that the data within a class have a normal distribution and a similar covariance matrix. If this assumption is violated (e.g., in the case of strongly non-linear relationships between variables), the effectiveness of this method may be limited. In the context of the studied samples, LDA may not provide a clear separation if the differences between classes are not sufficiently pronounced in the feature space [26].

This means that the model may not effectively capture the subtle influences of storage at different conditions, which is a limitation, especially if the features associated with these differences are not very pronounced in the data space.

The model used in the PCA and LDA provided valuable information in the context of synchronous fluorescence spectroscopy (SFS) data analysis. With PCA, it was possible to reduce the dimensionality of the large dataset, allowing the results to be presented for a few principal components that explained most of the variability in the data. This method allowed the identification of the main sources of spectral variability, such as differences between frozen and fresh samples and the effect of added sugar during pasteurisation. LDA, on the other hand, allowed a more effective distinction between sample groups, as it considered information on sample classes. It has helped us to better separate samples based on their belonging to different categories (e.g., fresh vs. frozen, pasteurised with sugar vs. pasteurised without sugar), which was particularly helpful in classifying and characterising the investigated products based on their spectral characteristics.

To the best of our knowledge, this is the first study applying spectrofluorimetric methods to assess quality changes in honeysuckle berry preserves. Compared to conventional chromatographic approaches, this method provides a rapid, non-destructive assessment of multiple compounds simultaneously, making it a promising tool for future food quality studies. The ability of PCA and especially LDA to differentiate between preservation methods and storage conditions demonstrates that spectrofluorimetry can serve as an efficient tool for monitoring quality changes in fruit-based products.

## 3. Materials and Methods

### 3.1. Plant Material

The fruits of honeysuckle (*Lonicera kamchatica*) were obtained from a local plantation. Preservation processes and fresh fruit analyses were performed no longer than 12 h after harvesting.

### 3.2. Honeysuckle Berry Preservation

The following variants of fruit preservation were used: pasteurised without sugar addition, pasteurised with sugar addition, and frozen.

Pasteurised samples were prepared as follows: a portion of 1 kg of fresh fruits was homogenised with simultaneous heating to 90 °C for 8 min using Thermomix (Vorwerk, Wuppertal, Germany), and the obtained pulp was filled into glass jars and pasteurised at 95 °C for 15 min. For pulp pasteurised with sugar, 400 g of sugar was used per 1 kg of fruit.

Fruits were frozen in the blast freezer (ZLN-T 300, Pol-Eko-Aparatura, Wodzisław Śląski, Poland), packed in foil bags, and then stored at −24 °C.

Pasteurised samples (with and without sugar) were divided into two parts and stored at 6 ± 1 °C and 22 ± 1 °C in the absence of light.

Analyses were carried out on the fresh fruits and samples just after preservation and after 1, 2, 3, 6, and 9 months of storage.

Samples of fruits (fresh and frozen after defrosting) were homogenised (Microtron MB 550, Kinematica AG, Malters, Switzerland), and pasteurised products were thoroughly mixed before filling the cuvette. The packaging of frozen products and the jars of pasteurised ones (three replicates) were chosen randomly from all accessible samples. Measurements were performed at a constant temperature of 22 °C.

### 3.3. Fluorescence Spectroscopy

Fluorescence spectra were registered using a Cary Eclipse spectrofluorimeter (Varian, Mulgrave, Victoria, Australia). A xenon lamp was used for excitation. Excitation and emission slit widths were 5 nm. Measurements were performed using a quartz cuvette with a 1 cm optical length in front-face mode using a solid sample holder (Varian). The incidence angle of the excitation radiation was set at 56°.

The fluorescence emission spectra were recorded in the wavelength range of 340–550 nm using an excitation wavelength of 280 nm. Synchronous fluorescence spectra were collected by simultaneously scanning the excitation and emission in the range of 240–750 nm with the constant Δλ between them. The spectra were recorded for Δλ in the 10–60 nm range every 10 nm. All spectra were registered in triplicate with a change in cuvette orientation at each measurement.

### 3.4. Statistical Analysis

Numerical analyses of fluorescence spectra were carried out using original procedures written in the R language (version 4.0.2), using FactoMineR 2.11 library packages [40] for PCA analysis and FactoExtra 1.07 [41] for the graphical presentation of the obtained results. Calculations of linear discriminants were performed using the original author’s code based on the MASS Package (MASS v.7.3-60) [42] and ggplot2 (ggplot2, v.3.5.1) [43] for the graphical presentation of the LDA results.

## 4. Conclusions

The preservation method used significantly affected the contents of the bioactive components analysed. An analysis of the fluorescence emission spectra of phenolic compounds showed differences in individual bioactive components’ decomposition rates, depending on the preservation method and storage conditions. During storage, the sugar added to part of the pasteurised samples stabilised some of the analysed compounds (gallic acid, p-coumaric acid, and phloridzin). Vitamins B_3_ and B_9_ were degraded by freezing to some extent, whereas their amounts in pasteurised samples (with and without sugar) were practically unchanged compared to fresh fruits. The opposite behaviour was observed in the case of chlorophylls. Freezing did not change the form and amount of the chorophylls present in the investigated samples, while pasteurisation caused the substantial degradation of the protochlorophylls.

PCA allowed confirmation of a small effect of the storage time and a moderate effect of added sugar for pasteurised samples. LDA made it possible to observe apparent differences between fresh, frozen, and pasteurised samples and to separate the analysed samples according to the storage conditions using subtle differences in fluorescence intensity observed in the registered synchronous spectra.

The advantages of the fluorimetric method used and the encouraging results of the presented qualitative analysis coupled with PCA and LDA provide the basis for trials of the further adaptation of this method, including a quantitative analysis of the quality of fruit preserves.

## Data Availability

The data are contained within the article.

## References

[1] Grobelna A., Kalisz S., Kieliszek M. (2019). Effect of Processing Methods and Storage Time on the Content of Bioactive Compounds in Blue Honeysuckle Berry Purees. Agronomy.

[2] Becker R., Szakiel A. (2019). Phytochemical Characteristics and Potential Therapeutic Properties of Blue Honeysuckle *Lonicera caerulea* L. (Caprifoliaceae). J. Herb. Med..

[3] Khoo H.E., Azlan A., Tang S.T., Lim S.M. (2017). Anthocyanidins and Anthocyanins: Colored Pigments as Food, Pharmaceutical Ingredients, and the Potential Health Benefits. Food Nutr. Res..

[4] Kucharska A.Z., Sokól-Lȩtowska A., Oszmiánski J., Piórecki N., Fecka I. (2017). Iridoids, Phenolic Compounds and Antioxidant Activity of Edible Honeysuckle Berries (*Lonicera caerulea* Var. *Kamtschatica sevast*). Molecules.

[5] Grobelna A., Kalisz S., Kieliszek M. (2019). The Effect of the Addition of Blue Honeysuckle Berry Juice to Apple Juice on the Selected Quality Characteristics, Anthocyanin Stability, and Antioxidant Properties. Biomolecules.

[6] Rupasinghe H.P.V., Arumuggam N., Amararathna M., De Silva A.B.K.H. (2018). The Potential Health Benefits of Haskap (*Lonicera caerulea* L.): Role of Cyanidin-3-O-Glucoside. J. Funct. Foods.

[7] Oszmiański J., Kucharska A.Z. (2018). Effect of Pre-Treatment of Blue Honeysuckle Berries on Bioactive Iridoid Content. Food Chem..

[8] Gorzelany J., Basara O., Kapusta I., Paweł K., Belcar J. (2023). Evaluation of the Chemical Composition of Selected Varieties of *L. caerulea* Var. *Kamtschatica* and *L. caerulea* Var. *Emphyllocalyx*. Molecules.

[9] Polak N., Kalisz S., Wawrzyńczak A., Kruszewski B. (2023). The Effect of Storage Time on Selected Quality Characteristics of Mixed Juices Made From Black Chokeberry and Honeysuckle Berry. Zywn. Nauk. Technol. Jakosc/Food. Sci. Technol. Qual..

[10] Piasek A., Kusznierewicz B., Grzybowska I., Malinowska-Pańczyk E., Piekarska A., Azqueta A., Collins A.R., Namieśnik J., Bartoszek A. (2011). The Influence of Sterilization with EnbioJet^®^ Microwave Flow Pasteurizer on Composition and Bioactivity of Aronia and Blue-Berried Honeysuckle Juices. J. Food Compos. Anal..

[11] Kalisz S., Kieliszek M. (2021). Influence of Storage Conditions on Selected Quality Characteristics of Blue Honeysuckle Berry Juice. Agrochimica.

[12] Jurgoński A., Juśkiewicz J., Zduńczyk Z. (2013). An Anthocyanin-Rich Extract from Kamchatka Honeysuckle Increases Enzymatic Activity within the Gut and Ameliorates Abnormal Lipid and Glucose Metabolism in Rats. Nutrition.

[13] Molina A.K., Vega E.N., Pereira C., Dias M.I., Heleno S.A., Rodrigues P., Fernandes I.P., Barreiro M.F., Kostić M., Soković M. (2019). Promising Antioxidant and Antimicrobial Food Colourants from *Lonicera caerulea* L. Var. *Kamtschatica*. Antioxidants.

[14] Šic Žlabur J., Colnar D., Voća S., Lorenzo J.M., Munekata P.E.S., Barba F.J., Dobričević N., Galić A., Dujmić F., Pliestić S. (2019). Effect of Ultrasound Pre-Treatment and Drying Method on Specialized Metabolites of Honeyberry Fruits (*Lonicera caerulea* Var. *Kamtschatica*). Ultrason. Sonochem..

[15] Orsavová J., Sytařová I., Mlček J., Mišurcová L. (2022). Phenolic Compounds, Vitamins C and E and Antioxidant Activity of Edible Honeysuckle Berries (*Lonicera caerulea* L. Var. *Kamtschatica pojark*) in Relation to Their Origin. Antioxidants.

[16] Wojdyło A., Jáuregui P.N.N., Carbonell-Barrachina Á.A., Oszmiański J., Golis T. (2013). Variability of Phytochemical Properties and Content of Bioactive Compounds in *Lonicera caerulea* L. Var. *Kamtschatica berries*. J. Agric. Food Chem..

[17] Kucharska A.Z., Fecka I. (2016). Identification of Iridoids in Edible Honeysuckle Berries (*Lonicera caerulea* L. Var. *Kamtschatica sevast*.) by UPLC-ESI-QTOF-MS/MS. Molecules.

[18] Zhang M., Ma X., Xiao Z., Sun A., Zhao M., Wang Y., Huang D., Sui X., Huo J., Zhang Y. (2023). Polyphenols in Twenty Cultivars of Blue Honeysuckle (*Lonicera caerulea* L.): Profiling, Antioxidant Capacity, and α-Amylase Inhibitory Activity. Food Chem..

[19] Hegazi N.M., Elghani G.E.A., Farag M.A. (2022). The Super-Food Manuka Honey, a Comprehensive Review of Its Analysis and Authenticity Approaches. J. Food Sci. Technol..

[20] Sikorska E., Khmelinskii I.V., Sikorski M., Caponio F., Bilancia M.T., Pasqualone A., Gomes T. (2008). Fluorescence Spectroscopy in Monitoring of Extra Virgin Olive Oil during Storage. Int. J. Food Sci. Technol..

[21] Cao J., Li C., Liu R., Liu X.R., Fan Y., Deng Z.Y. (2017). Combined Application of Fluorescence Spectroscopy and Chemometrics Analysis in Oxidative Deterioration of Edible Oils. Food Anal. Methods.

[22] Domínguez Manzano J., Muñoz de la Peña A., Durán Merás I. (2019). Front-Face Fluorescence Combined with Second-Order Multiway Classification, Based on Polyphenol and Chlorophyll Compounds, for Virgin Olive Oil Monitoring Under Different Photo- and Thermal-Oxidation Procedures. Food Anal. Methods.

[23] Sikorska E., Włodarska K., Khmelinskii I. (2020). Application of Multidimensional and Conventional Fluorescence Techniques for Classification of Beverages Originating from Various Berry Fruit. Methods Appl. Fluoresc..

[24] Karoui R., Blecker C. (2011). Fluorescence Spectroscopy Measurement for Quality Assessment of Food Systems—A Review. Food Bioprocess Technol..

[25] Sádecká J., Tóthová J. (2007). Fluorescence Spectroscopy and Chemometrics in the Food Classification—A Review. Czech J. Food Sci..

[26] Efenberger-Szmechtyk M., Nowak A., Kregiel D. (2018). Implementation of Chemometrics in Quality Evaluation of Food and Beverages. Crit. Rev. Food Sci. Nutr..

[27] Karoui R., Dufour E., Bosset J.O., De Baerdemaeker J. (2007). The Use of Front Face Fluorescence Spectroscopy to Classify the Botanical Origin of Honey Samples Produced in Switzerland. Food Chem..

[28] Lang M., Stober F., Lichtenthaler H.K. (1991). Fluorescence Emission Spectra of Plant Leaves and Plant Constituents. Radiat. Environ. Biophys..

[29] Ali H., Khan S., Ullah R., Khan B. (2020). Fluorescence Fingerprints of Sidr Honey in Comparison with Uni/Polyfloral Honey Samples. Eur. Food Res. Technol..

[30] Lakowicz J., Lakowicz J. (2006). Principles of Fluorescence Spectroscopy.

[31] Vidot K., Devaux M.F., Alvarado C., Guyot S., Jamme F., Gaillard C., Siret R., Lahaye M. (2019). Phenolic Distribution in Apple Epidermal and Outer Cortex Tissue by Multispectral Deep-UV Autofluorescence Cryo-Imaging. Plant Sci..

[32] Radotić K., Stanković M., Bartolić D., Natić M. (2023). Intrinsic Fluorescence Markers for Food Characteristics, Shelf Life, and Safety Estimation: Advanced Analytical Approach. Foods.

[33] Barbieri D., Gabriele M., Summa M., Colosimo R., Leonardi D., Domenici V., Pucci L. (2020). Antioxidant, Nutraceutical Properties, and Fluorescence Spectral Profiles of Bee Pollen Samples from Different Botanical Origins. Antioxidants.

[34] Parri E., Santinami G., Domenici V. (2020). Front-Face Fluorescence of Honey of Different Botanic Origin: A Case Study from Tuscany (Italy). Appl. Sci..

[35] Jurikova T., Rop O., Mlcek J., Sochor J., Balla S., Szekeres L., Hegedusova A., Hubalek J., Adam V., Kizek R. (2012). Phenolic Profile of Edible Honeysuckle Berries (Genus Lonicera) and Their Biological Effects. Molecules.

[36] Sikorska E., Khmelinskii I., Sikorski M., Boskou D. (2012). Analysis of Olive Oils by Fluorescence Spectroscopy: Methods and Applications. Olive Oil—Constituents, Quality, Health Properties and Bioconversions.

[37] Qin Y., Chen X., Xu F., Gu C., Zhu K., Zhang Y., Wu G., Wang P., Tan L. (2023). Effects of Hydroxylation at C3′ on the B Ring and Diglycosylation at C3 on the C Ring on Flavonols Inhibition of α-Glucosidase Activity. Food Chem..

[38] Mukaida N., Kawai N., Onoue Y., Nishikawa Y. (1993). Three-Dimensional Chromatographic Analysis of Protochlorophylls in the Inner Seed Coats of Pumpkin. Anal. Sci..

[39] Naziri E., Mitić M.N., Tsimidou M.Z. (2016). Contribution of Tocopherols and Squalene to the Oxidative Stability of Cold-Pressed Pumpkin Seed Oil (*Cucurbita pepo* L.). Eur. J. Lipid Sci. Technol..

[40] Le S., Josse J., Husson F. (2008). FactoMineR: An R Package for Multivariate Analysis. J. Stat. Softw..

[41] Kassambara A., Mundt F. Factoextra: Extract and Visualise the Results of Multivariate Data Analyses. R Package Version 1.0.999. http://www.sthda.com/english/rpkgs/factoextra.

[42] Kafadar K., Koehler J.R., Venables W.N., Ripley B.D. (1999). Modern Applied Statistics with S-Plus.

[43] Wickham H. (2016). Ggplot2: Elegant Graphics for Data Analysis.

